# Effectiveness of Interventions to Reduce Contact Rates during a Simulated Influenza Pandemic

**DOI:** 10.3201/eid1304.060828

**Published:** 2007-04

**Authors:** Michael J. Haber, Davis K. Shay, Xiaohong M. Davis, Rajan Patel, Xiaoping Jin, Eric Weintraub, Evan Orenstein, William W. Thompson

**Affiliations:** * Emory University Rollins School of Public Health, Atlanta, Georgia, USA; † Centers for Disease Control and Prevention, Atlanta, Georgia, USA; ‡ Amgen, Inc., Thousand Oaks, California, USA; § Yale University, New Haven, Connecticut, USA

**Keywords:** influenza, models, statistical, patient isolation, quarantine, stochastic processes, research

## Abstract

Measures to decrease contact between persons during an influenza pandemic have been included in pandemic response plans. We used stochastic simulation models to explore the effects of school closings, voluntary confinements of ill persons and their household contacts, and reductions in contacts among long-term care facility (LTCF) residents on pandemic-related illness and deaths. Our findings suggest that school closings would not have a substantial effect on pandemic-related outcomes in the absence of measures to reduce out-of-school contacts. However, if persons with influenzalike symptoms and their household contacts were encouraged to stay home, then rates of illness and death might be reduced by ≈50%. By preventing ill LTCF residents from making contact with other residents, illness and deaths in this vulnerable population might be reduced by ≈60%. Restricting the activities of infected persons early in a pandemic could decrease negative health impact.

Three influenza pandemics have occurred during the 20th century (in 1918, 1957, and 1968), and another pandemic is inevitable (*1*). The requirements for a pandemic virus include the existence of a new influenza A hemagglutinin for which there is little immunity, the ability of this strain to infect humans efficiently, and person-to-person transmission. Such viruses are likely to arise in densely populated agricultural communities where contact between humans and birds or pigs are close and persistent (*2*). In 1997, a highly pathogenic avian influenza A (H5N1) virus was transmitted from live poultry to humans in Hong Kong Special Administrative Region, People’s Republic of China, killing 6 of 18 infected persons (*3*). From December 2003 through June 6, 2006, the World Health Organization confirmed 225 human cases and 128 deaths associated with influenza A (H5N1) infections in humans (*4*), and in October 2005, influenza A (H5N1) infections among birds were identified for the first time in Europe. Currently circulating influenza A (H5N1) viruses appear to infrequently infect humans, and person-to-person transmission, if it occurs, is certainly not efficient. However, international health officials are concerned that, as human exposure to such viruses increases, so does the possibility that a pandemic virus might appear.

The next influenza pandemic in the United States could result in 89,000 to 207,000 deaths, 314,000 to 734,000 hospitalizations, and 18 to 42 million outpatient visits, with a direct economic effect between US $71 and $166 billion, according to 1 set of estimates (*5*). Others have described the possible effects of vaccine and antiviral interventions. One study estimated that vaccinating 60% of the population would be necessary to achieve optimal cost benefits, assuming that development and mass production of a vaccine would require 6–8 months after the pandemic virus was characterized (*5*). Longini et al. (*6*) estimated the effectiveness of rapid targeted antiviral prophylaxis of persons early in a pandemic by using epidemic stochastic simulations. They found that if the next pandemic virus had a similar virulence to that of the 1957–58 pandemic virus, then delivering prophylaxis to 80% of exposed persons for up to 8 weeks could reduce attack rates by 2%–33% and death rates by 0.04–0.58/1,000 persons. However, such a strategy would require a stockpile of 1.9 billion doses of antiviral agents, which exceeds the current production capacity for these drugs for at least the next 5 years.

In the absence of adequate supplies of vaccines and antiviral agents, at least during the first wave of an influenza pandemic, public health officials should consider using interventions designed to reduce the number of contacts between infected or exposed persons and susceptible persons. The US Department of Health and Human Services Influenza Pandemic Plan discusses several possible containment strategies, including those directed to single persons or entire communities (*7*). We used new stochastic simulation models to estimate the effects of several interventions of this kind. These models represented the spread of a pandemic in an urban US community, allowing for contacts in different settings (or mixing groups), including households, daycare centers, schools, workplaces and long-term care facilities (LTCFs). By using the age distribution of the US population (*8*), we placed each person in the community in a stratum, defined by age group and (if ≥65 years of age) by residence in the community or in an LTCF. Person-to-person transmission probabilities depended on the daily duration of contacts. Contact rates and their duration varied by each person’s stratum and mixing groups. By using these models to simulate an influenza pandemic, we estimated the effects of school closings, home confinement of ill persons (i.e., isolation) or their household contacts (i.e., quarantine), and reduction of contacts among residents of LTCFs on overall illness attack rates, hospitalization rates, and mortality rates.

## Materials and Methods

### Simulation Model

We simulated an influenza outbreak in a small urban US community. The simulation model used data from the Asian influenza A (H2N2) pandemic in 1957–58 (*6*) and from studies on US influenza-related excess rates of hospitalizations of illness and death (*9**–**11*). The simulation process begins with the generation of a community of households, where the distributions of sizes of the households and ages of the household members follow the 2000 US Census. Every person in the community belongs to 1 of 5 age-dependent strata: preschool children (ages birth–4 years), schoolchildren (ages 5–18 years), adults (ages 19–64 years), seniors (ages ≥65 years) living at home, and seniors (ages ≥65 years) living in an LTCF. In addition, each person belongs to ≥1 mixing groups, according to his or her stratum: households, daycare centers, schools, workplaces, LTCFs, and the community. The mixing matrix is presented as Table 1.

**Table 1:** Mixing matrix for the simulation model

Age stratum, y	Mixing group					
	Household	Daycare center	School	Workplace	Community	LTCF*
0–4	+	+			+	
5–18	+		+		+	
19–64	+			+	+	
≥65, at home	+				+	
≥65, in LTCF						+

*LTCF, long-term care facility.

On any given day, a susceptible person, A, makes contacts with other persons that may lead him or her to become infected. These contacts take place in each of A’s mixing groups. The probability that person A becomes infected depends on the following input parameters: 1) the number of different persons with whom person A has contact in each mixing group, 2) the total duration, in minutes, of all the contacts with each of these persons, and 3) the per-minute rates of infection transmission if the contacted person is infectious. The number and duration of contacts may be different on weekdays and weekend days. The values of the parameters that were used in this study are presented in the Appendix. Once person A becomes infected, he or she undergoes a latent period, followed by a period in which he or she is infectious. The mean length of the latent and infectious periods are input parameters.

This model has 3 new features that are not shared by the commonly used simulation models (such as the model in [6]) for transmission of influenza: 1) the probability of transmission depends on the total duration of all contacts between 2 persons, rather than on the number of times they make a contact, 2) the transmission parameters do not depend on the population size, and 3) different contact parameters can be specified for weekdays and weekend days. Technical details of the simulation model are presented in the Appendix. The basic reproductive number (R_0_) for this model is 2.7. This value is within the range (2.0–3.0) estimated by Mills et al. (*12*) for the 1918 influenza pandemic.

### Interventions

The interventions we examined in this simulation study were school closings, confinement of ill persons and their household contacts to their homes, and reduction in contact rates among residents of LTCFs. Interventions were implemented at the start of the outbreak.

#### School Closings

When this intervention was implemented, schools closed when the prevalence of illness among children in the school exceeded a predetermined threshold, set to 10%, 15%, or 20% in the simulations. A school remained closed for a predetermined period (7, 14, or 21 days). On weekdays, household and community contact parameters of children whose school was closed were assigned their weekend levels; their contacts with other children who continued to attend school and with working adults did not change.

#### Confinement to Home

When this intervention was implemented, a given fraction of households were assumed to comply. If a household complied, then all of its members followed the confinement rules unless they had been previously ill and had recovered. We considered 2 types of confinement: ill persons only, and ill persons and all the members of the same household. Confinement began after a given number of days of illness (1, 2, or 3 days) and did not depend on the severity of illness. If symptoms were severe, then the person reduced his or her duration of contacts with other household members by 50%.

When a person was confined on a weekday (because of his or her illness or illness of another household member) and did not withdraw due to severe symptoms, then the duration of contacts with household members who continued to go to school or work did not change. Durations of contacts with household members who stayed at home and were not withdrawn were the same as on a weekend day.

When ill persons were confined, they returned to school or work 1 day after their illness ended. When ill persons and other household members were confined, a person returned to school or to work 1 day after his or her illness ended (even if other ill persons remained in the household). A person who did not become ill returned to school or work on the third day after the last day of illness of any household member (because the length of the latent/incubation period was assumed to be 2 days).

#### Reduction of Contacts in LTCFs

We examined the effects of 2 interventions on LTCF residents: reduction in duration of contacts with other residents who were ill, and reduction in duration of contacts with visiting family members. Contacts with LTCF staff did not change.

### Effectiveness of Interventions

We first ran a set of 200 simulations using the baseline settings for all the parameters, without any interventions (Appendix). The average rates for the 3 outcomes of interest—overall illness rate, hospitalization rate, and death rate—were calculated for 200 simulations and used as baseline rates. For each intervention, we ran a set of 200 simulations and used the averages of these simulations as estimates of the expected rates under this intervention. The effectiveness of each intervention was defined as follows:

Effectiveness = [(baseline rate) – (rate with intervention)]/baseline rate

### Sensitivity Analysis

We performed a sensitivity analysis to assess the robustness of our findings regarding the effectiveness of the 3 modeled interventions. In common with all simulation studies, our findings depended on several parameters for which we have estimated values that we believe are reasonable starting points. These values included baseline contact rates, the probability of illness given infection, the relative infectiousness of an infected person without influenza symptoms, the probability of withdrawal to home because of severe symptoms, and the reduction in contact rates due to severe symptoms. We varied the values of these parameters and examined the effects of these changes on estimates of the effectiveness of school closings and confining ill persons to their homes.

## Results

### Baseline Rates

Based on the 200 simulations conducted with the baseline values of the pandemic parameters, the baseline rate of illness was 32.1%, (95% confidence interval [CI] 31.2%–32.9%), the baseline rate of hospitalization was 196.9/100,000 (95% CI 183.2–210.6) and the baseline rate of death was 63.4/100,000 (95% CI 56.2–70.6). These results were based on the assumption that the illness rates would be similar to their values in the 1957 influenza pandemic.

### School Closings

**Figure 1:**
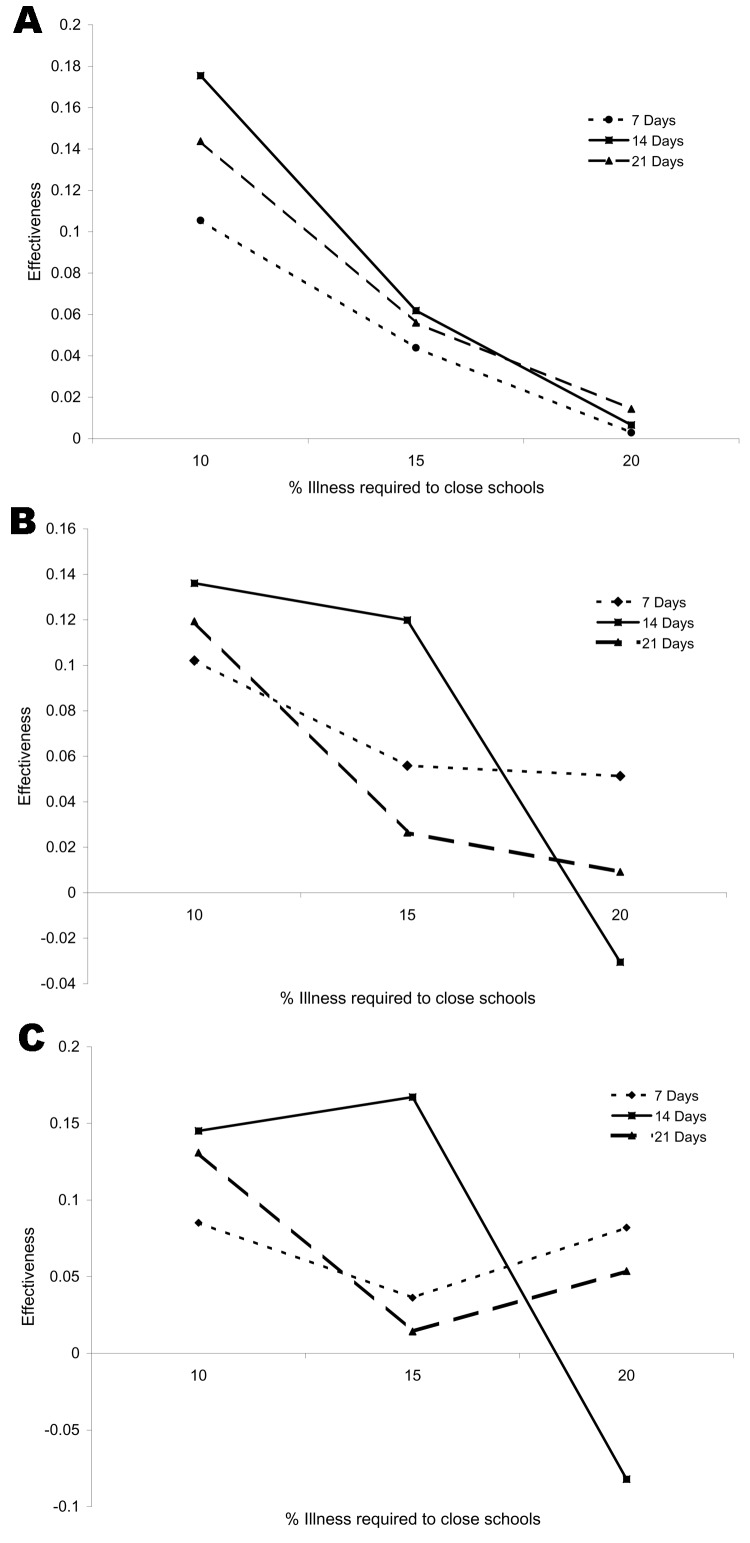
Estimated effectiveness of closing schools on illness (A), hospitalization (B), and death (C) rates during a simulated pandemic.

**Figure 2:**
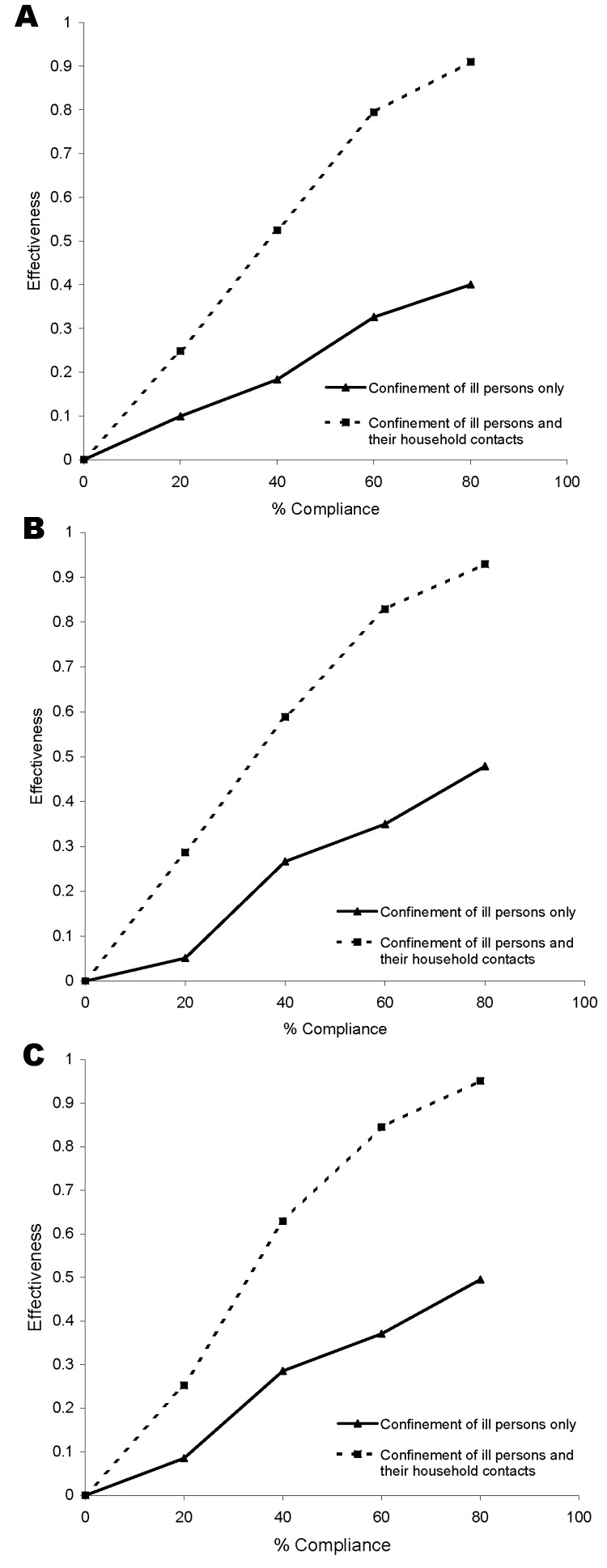
Estimated effectiveness of confinement to home 2 days after onset of respiratory symptoms on illness (A), hospitalization (B), and death (C) rates during a simulated pandemic.

Two parameters affected the effectiveness of school closings: the percentage of ill schoolchildren required to close a school and the number of days the school remained closed. The effectiveness of the intervention varied as a function of the percentage of ill persons required for closing a school and the duration of the closure (Figure 1). For example, if each school were closed for 7 days when the proportion of ill children exceeded 10%, then the overall illness rate was 0.288 (95% CI 0.278–0.297). The baseline illness rate was 0.321; therefore, the effectiveness of this intervention was (0.321–0.288)/0.321 = 0.103 (95% CI 0.075–0.131). As expected, effectiveness usually decreased as the percentage of ill children required to close a school increased. The effect of the length of closure was less clear (Figure 2). When schools were closed, transmission in households and in the community increased; thus, school closings could increase death and illness rates in some groups. For example, when the illness rate required for school closing was 10%, then closing schools for 14 days had the largest effect on hospitalization rates, compared with closings of 7 or 21 days. However, when the rate for closing was 20%, then closing schools for 14 days had a smaller effect on hospitalization rates than closing for 7 or 21 days.

### Confinement to Home

In our models, confinement to home took place after a person showed symptoms of influenza. A delay of 1, 2, or 3 days occurred between onset of symptoms (which coincided with the onset of infection) and the beginning of the confinement period. This delay and the proportion of households that complied with the confinement rules affected the effectiveness of the intervention. Figure 2 presents the effectiveness of these interventions as a function of the percentage of households that comply (between zero and 80%) for a delay of 2 days. As expected, effectiveness usually increased with the compliance percentage. Confining the ill persons and their household members was more effective than confining the ill persons only. For example, given a delay of 2 days and 60% compliance, the effectiveness of these interventions on illness rates was 0.33 for confining the ill only and 0.80 for confining ill persons and their household members. Effectiveness decreased when the length of the delay was increased.

### Reducing Contacts in LTCFs

Reducing contacts with ill residents of LTCFs decreased the rates of illness, hospitalization, and death for LTCF residents by >50% (Table 2). Reducing contacts also decreased the rates of hospitalization and death in the general population by up to 14% and 24%, respectively.

**Table 2:** Estimated effects of pandemic interventions in long-term care facilities (LTCFs) on illness, hospitalization, and death rates

Outcome rates	Rates for general population			Rates for LTCF residents		
	Illness	Hospitalization	Death	Illness	Hospitalization	Death
Reduction in contacts with ill residents (%)						
25	0.02*	0.10	0.14	0.22	0.32	0.33
50	0.04	0.13	0.23	0.37	0.44	0.41
75	0.04	0.14	0.24	0.54	0.55	0.59
100	0.03	0.14	0.21	0.65	0.60	0.60
Reduction in contacts with visitors (%)						
25	0.01	0.11	0.12	−0.02	0.03	−0.03
50	0.02	0.06	−0.02	0.03	−0.05	−0.05
75	0.04	0.15	0.20	0.00	0.05	−0.03
100	0.04	0.07	0.12	0.03	0.11	0.11

*Thus, a 25% reduction in contacts with ill residents of LTCFs was estimated to reduce the illness rate for the population by 2% and the illness rate for LTCFs by 22%.

### Effect of Intervention on Dynamics of the Pandemic

**Figure 3:**
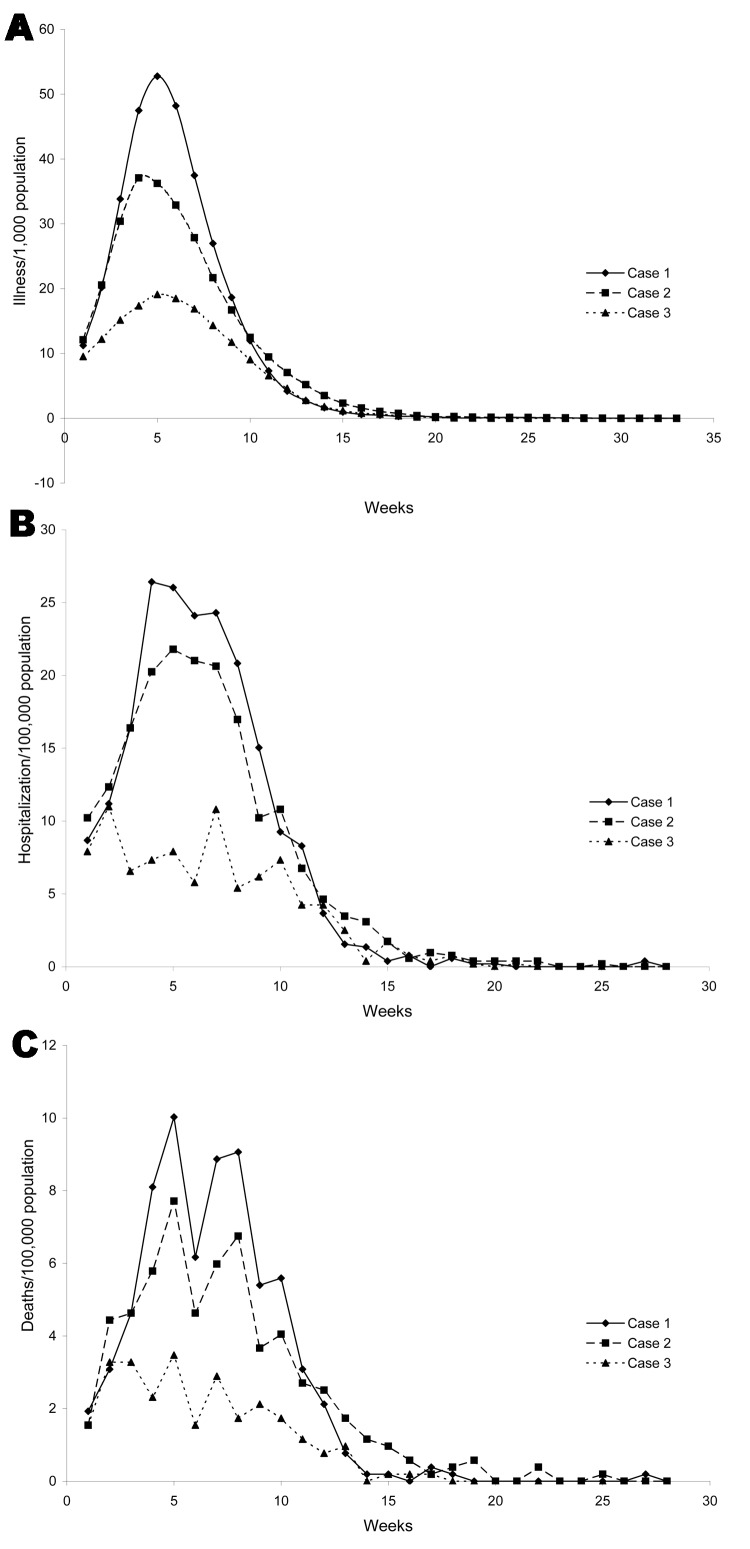
Dynamics of the influenza pandemic. Case 1: no interventions. Case 2: schools are closed for 14 days when prevalence reaches 10%. Case 3: ill persons and all their household contacts are confined to their homes after the second day of illness of the index case-patient, and the compliance rate is 40%. A) illness; B) hospitalizations; C) deaths.

Figure 3 presents the dynamics of the pandemic (a) without any intervention, (b) when schools are closed for 14 days as the proportion of ill children exceed 10%, and (c) when ill persons and all their household contacts are confined after the second day of illness of the index case-patient and compliance is 40%. We see that these interventions do not affect the time to the peak of the pandemic (around week 5). The rate of decline following the peak does not change under confinement to home, while it slightly decreases under school closing.

### Sensitivity Analysis

The value of the basic reproductive number (R_0_) for the baseline setting of our parameters is 2.7. Because this value is higher than values used in recent simulation studies (*13**,**14*), we evaluated the effectiveness of the interventions under smaller values of R_0_. We found that reducing R_0_ resulted in an increase in the effectiveness of confinement to home and a decrease in the effectiveness of school closings. Thus, our findings regarding the effectiveness of confinement and the lack of effectiveness of school closings remain valid for smaller values of R_0_. The results of additional sensitivity analyses were as follows.

#### School Closings

The most important parameters related to the effectiveness of school closings are those that underlie the contacts between children while they are in school. In our simulations we assumed that on a school day each child makes contact with 10 other schoolchildren, each contact lasting 120 minutes (see section D.1.a in the Appendix). Some of these contacts may be concurrent. To examine the effect of changing each child’s exposure to other schoolchildren on the effectiveness of school closures, we increased and decreased the baseline duration of 120 minutes by 50%. Table 3 shows the effectiveness of closing schools for 14 days for the 3 baseline values of duration of school contact. As we see, longer or shorter durations of contact while schools are open do not result in substantial changes in the effectiveness of school closings.

**Table 3:** Effect of baseline contact durations in school on effectiveness of closing schools for 14 days

School baseline contact duration, min	% Ill for school closing	% Effectiveness		
		Illness rate	Hospitalization rates	Heath rates
120	10	17	14	14
180	10	17	16	20
60	10	12	5	6
				
120	15	6	12	17
180	15	8	8	12
60	15	3	1	−13
				
120	20	1	−3	−8
180	20	3	4	5
60	20	−1	0	−8

#### Confinement to Home

We varied the values of several parameters in the baseline model and examined the effects these changes had on estimates of the effectiveness of confinement of ill persons to their homes (Table 4). We assumed that 40% of ill persons without severe symptoms were confined to home within 2 days of symptom onset. When the fraction of infected persons who developed symptoms was increased from 0.67 to 0.93, then the illness rate without an intervention (i.e., at the baseline level) changed only from 0.333 to 0.319, while implementation of the intervention changed this rate from 0.272 to 0.242. Thus, the effectiveness of this intervention increased from 0.183 to 0.241. The alternative values we used in Table 2 modeled a more severe pandemic than the pandemic modeled with the baseline initial values.

**Table 4:** Effectiveness of confinement of ill persons to their homes, with a 2-d delay and 40% compliance, for differing values of the initial parameters

Parameter	% Effectiveness		
	Illness rates	Hospitalization rates	Death rates
Rate of withdrawal due to severe symptoms (children/adults)			
0.75*/0.50*	0.18	0.27	0.29
0.55/0.30	0.34	0.40	0.41
Relative contact duration when withdrawn due to severe symptoms			
0.50*	0.18	0.27	0.29
0.70	0.14	0.21	0.19
Fraction of infected persons having symptoms			
0.67*	0.18	0.27	0.29
0.93	0.24	0.24	0.27
Relative infectiousness of non-ill persons			
0.50*	0.18	0.27	0.29
0.70	0.19	0.24	0.28

*Values used in the baseline simulation models.

## Discussion

The continuing epizootic of influenza A (H5N1) among birds in Asia and Europe has raised concerns that the likelihood of an influenza pandemic may be increasing. Shortages in the supply of neuraminidase inhibitors, the antiviral agents most likely to be effective against a pandemic influenza strain, and the months needed from the isolation of a pandemic strain until the availability of vaccine suggest that reducing contact rates between infected and uninfected persons will represent one of the few sets of interventions that can be rapidly implemented. We used a stochastic simulation model to estimate the effectiveness of several interventions that could reduce contact rates on pandemic-related outcomes.

The Pandemic Influenza Strategic Plan and Public Health Guidance for State and Local Partners prepared by the US Department of Health and Human Services was released on November 2, 2005 (*7*). This plan discusses the use of individual-level (e.g., isolation and quarantine) and community-level (e.g., school closings) containment measures. Our study considered possible interventions of both kinds, including early identification and confinement of case-patients and their household contacts, limiting visits to LTCFs, and closing of schools.

Our findings suggest that closing schools would result in relatively small reductions in morbidity and mortality rates during a pandemic. For example, when schools were closed when ≥10% of children had influenza symptoms and remained closed for 14 days, the rates of illness, hospitalization, and death decreased from the baseline rates of 32.1%, 197/100,000, and 63/100,000 to 26.5%, 170/100,000, and 54/100,000, respectively. Thus, the effectiveness of school closings was ≈14%–18%. When we increased the threshold of illness incidence required for school closing to 20%, then these rates were 31.9%, 203/100,000, and 69/100,000, respectively. These mild decreases in the rates of illness and death after school closures are explained by the fact that in our models, children whose schools were closed were more likely to increase their contacts with other groups. The attack rate of 62% that we used for school-age children may be considered high. However, if the attack rate were reduced, school closings would have an even smaller effect. Our results do not contradict recent findings that vaccination of schoolchildren could be effective in controlling transmission during a seasonal influenza epidemic (*15*). Vaccination of children reduces their chances of infection and of transmitting infection to household and community contacts, whereas closing schools may not decrease the likelihood of infection substantially and could increase the probability that an infected child will infect household and community contacts (*14*).

The effect of school closings on overall illness rates in an influenza pandemic has been estimated in other recent simulation studies. Germann et al. (*16*) modeled the effect of a pandemic on the entire US population. They found that for R_0_ ≥1.9, closing of schools without any additional interventions had limited effectiveness. On the other hand, for R_0_ ≤1.6, school closings reduced the extent of illness. Carrat et al. (*17*), by using a simulation model for the spread of influenza in a community, found school closings to be effective. We believe that these inconsistencies in the reported effects of school closings depend on the details of the various simulation models, especially on the way the community is affected by school closing in terms of increased contact rates of schoolchildren when their school is closed.

Our simulations predict that it might be possible to decrease illness and death rates by as much as 50% by reducing the contact rates of all ill persons. However, achieving this level of effectiveness would require persuading 60% of those with symptoms to withdraw to their homes and confine themselves. Simulation studies by Longini et al. (*13*) and Ferguson et al. (*14*) found that quarantine, when used in conjunction with vaccines and antiviral agents, would be effective in containing an influenza pandemic in Southeast Asia. One should remember that the effectiveness of any behavioral/social intervention may vary across cultures.

Residents of LTCFs are likely to be at high risk for serious pandemic-related illness and death. We found that by limiting contacts of ill residents, illness and death may be reduced among other residents. These are notable findings, as this vulnerable population responds poorly to seasonal influenza vaccination, and they are unlikely to receive the limited quantities of pandemic vaccine when it first becomes available.

The effectiveness of any particular intervention designed to reduce contact rates depends on the initial values selected for the parameters affecting influenza transmission (e.g., contact durations, probability of withdrawal due to severe symptoms), and a limitation of our study is that few data exist on which to base these values. Studies designed to obtain reliable estimates of these parameters during seasonal, interpandemic influenza outbreaks should be a high priority. However, the major findings of this study seem to be robust, given a range of realistic values for the parameters we used. The target attack rates we used to calibrate the contact parameters (provided in the Appendix) are high, but lowering these attack rates should not have a major effect on our findings, because both the pre- and postintervention incidence rates would decrease concomitantly.

We did not make formal estimates of the economic costs and benefits of the interventions we examined. However, some likely consequences of school closings may be considered, given current childcare practices. Obviously, the longer the duration of school closure, the more costly the consequences as working parents either have to take time off work to supervise children or pay for somebody else to care for them. If a large number of school days are lost, school districts might consider extending the school year, which would incur additional costs, although the conditions would be expected to vary greatly between school districts. These increased costs would have to be weighed against the limited predicted effectiveness of this intervention. Encouraging the voluntary withdrawal of ill persons appears to be a more effective strategy than school closings in reducing the impact of a pandemic, and it may represent a relatively inexpensive intervention. However, researchers have found that US workers routinely miss <1 day of work after reporting onset of influenzalike illness (*18*). Encouraging longer durations of work loss could decrease compliance with self-isolation and increase the economic cost per case avoided. Home quarantine of the immediate family members of an ill person would likely increase the costs per case averted. For example, during the quarantine efforts related to the severe acute respiratory syndrome outbreak in Toronto (*19*), many families found it too expensive to rigidly comply with a household-level quarantine of ≥7 days.

Our stochastic simulation model has several strengths. The model considers the length of time 2 persons are in contact, in addition to the total number of contacts. The model parameters we used are not related to the size of the simulated population, unlike previous models (*6*). We repeated the simulations conducted for this study with a population twice as large as the original population and the same input parameters. The resulting rates were almost unchanged, so the differences can be attributed to the random effects associated with these simulations. The weaknesses of our present model are that it requires many input parameters and that it does not include the effects of antiviral medications. Our model allows for estimating vaccine effects for susceptibility and infectiousness; however, this option was not used in the present study. On February 1, 2007, the Centers for Disease Control and Prevention (CDC) issued an Interim Pre-Pandemic Planning Guidance: Community Strategy for Pandemic Influenza Mitigation in the United States (*20*). This document recommends several nonpharmaceutical interventions during a severe pandemic, including isolation of persons with confirmed or probable influenza, voluntary home quarantine of members of households with confirmed cases, dismissal of students from schools and school-based activities, and closure of childcare programs. During a pandemic with a severity index of 4 or 5 (defined as a case fatality rate of >1%), this new guidance recommends not only school dismissals of ≤12 weeks but also measures to protect children from being exposed or exposing others to the pandemic virus via reduction of their out-of-school social contacts and community mixing. In this article, we assessed the effectiveness of school closures of 1–3 weeks duration after school absenteeism rates reached high levels. We assumed that children dismissed from schools would increase their out-of-school contacts. These assumptions reduced the effectiveness of school closures in our model. In future work, we will explore the effectiveness of early dismissal of students from schools, together with changes in out-of-school contacts, and other interventions using our model.

In summary, if persons who suspect they are infected with pandemic influenza virus were to withdraw to their homes quickly, the rates of illness and death associated with a pandemic may be substantially reduced. The withdrawal of all household contacts may further reduce rates of illness and death, but this additional intervention is likely to be relatively costly and difficult to implement. Restricting the movement of ill LTCF residents will be beneficial in reducing their adverse health outcomes. Before early and rapid implementation of such interventions during a pandemic is feasible, the public will need to be educated about the early symptoms of influenza and measures developed to increase the social acceptability of self-isolation when ill.

## Supplemental Materials Appendix

### Details of the Simulation Model

#### The Model

The following parameters describe how persons made contact with others. For individual *A* from stratum *i*_A_ and mixing group of type *k* and stratum *j*, we denoted by Ψ *_Ajkl_* the group of all persons with whom *A* made contact on a day of type *l* , where *l* = 1 for weekdays and *l* = 2 for weekend days. These groups were referred to as "contact groups." The size, *^c^_iAjkl_* , of Ψ *_Ajkl_* and the average total duration in minutes, *^d^_iAjkl_*, of all the contacts made by *A *with each member of Ψ *_Ajkl_* on 1 day were specified as input parameters. At the beginning of each simulation, the initial contact groups Ψ *_Ajkl_* were determined for *A* by selecting at random *^c^_iAjkl _*persons of stratum *j* from each mixing group other than the household to which *A*belonged. For households (*k* = 1) the contact groups consisted of all household members (other than *A*) in the corresponding stratum. If a mixing group had fewer than *^c^_iAjkl _* members of stratum *j*, then the contact group consisted of the entire mixing group.

Influenza transmission was determined by contact parameters and transmission rates. The rate of viral transmission per minute of contact from an infected person in stratum *j* to a susceptible person in stratum *i* (where *i*, *j = 1,2,3,4,5*) was denoted by *λ_ϋ_*. The probability that transmission occurred during a contact of *d* minutes was 1 –exp{–*λ_ϋ_d*}. On each day of the simulated outbreak, the model calculated for each susceptible person the probability of becoming infected that day, based on the contacts made with all persons in each contact group. Consider a susceptible person *A*from stratum *i_A_* and a person *B* in one of *A*'s contact groups, Ψ *_Ajkl_* . Define *^y^_B_* = 0 if *B* was not infectious and *^y^_B_* = 1 if *B* was infectious. The probability that *A* escaped infection from *B* that day was exp {–*λ_ϋ_d_i Ajkl_^y^_B_*}. To remain uninfected, *A *must have escaped infection from all the members of all her/his contacts groups. Hence the probability that *A *became infected on this day was: *P*(inf) = 1 - П*_k_*П*_j_*П*ΒЄΨ_Ajkl_* exp{–λ_ϋ_*d_iAjkl_^y^_B_*}. This probability was compared with a random number, *r*, drawn from the interval [0,1]. The person *A* became infected if *r* < *P*(inf).

Each newly infected person entered a latent period, at the conclusion of which the person became infectious to others, based on values estimated by Elveback et al. (*21*). We assumed that the probability of symptoms developing, given influenza infection, was 0.67 and that an infected person who did not become ill was 50% less infectious than one who did. An ill person with severe symptoms withdrew to home, made contacts only with household members, and the duration of these contacts was decreased by 50%. We assumed that in 50% of adults and 75% of children severe symptoms developed and the person withdrew. An ill person could require hospitalization or die from influenza complications. The probabilities of hospitalization and death were determined on the basis of the distribution of age-specific hospitalizations and deaths in an average seasonal (nonpandemic) influenza season (*22**–**24*) and on the total hospitalization and death rates expected in a pandemic that is similar to the Asian influenza pandemic of 1957–58, for which the overall illness attack rate was estimated at 0.33, with an influenza death rate of 0.58/1,000 persons (*25*). A list of the initial settings of all the parameters used in these models is provided below.

The simulated epidemic started with a small number of infective persons. The transmission process continued until no further infected persons remained in the community. At the end of each simulated epidemic, the program determined the proportion of persons who became ill as well as the proportions of hospitalizations and deaths in the community. We ran 200 simulations and calculated the means of the above 3 proportions over these simulated epidemics.

### Baseline Values of Parameters

#### **A. Influenza-related Parameters (based on Longini et al. [** 25 **])**

The following parameters describe how persons made contact with others. For individual *A* from stratum *i_A_* and mixing group of type *k* and stratum *j*, we denoted by Ψ *_Ajkl_* the group of all persons with whom *A* made contact on a day of type *l*, where l = 1for weekdays and *l* = 2 for weekend days. These groups were referred to as "contact groups." The size, *^c^_iAjkl_*, of Ψ *_Ajkl_* and the average total duration in minutes, *^d^_iAjkl_*, of all the contacts made by *A* with each member of Ψ *_Ajkl_* on 1 day were specified as input parameters. At the beginning of each simulation, the initial contact groups Ψ *_Ajkl_* were determined for *A* by selecting at random *^c^_iAjkl_* persons of stratum *j* from each mixing group other than the household to which *A* belonged. For households (*k* = 1) the contact groups consisted of all household members (other than *A*) in the corresponding stratum. If a mixing group had fewer than *^c^_iAjkl_* members of stratum *j*, then the contact group consisted of the entire mixing group.

Influenza transmission was determined by contact parameters and transmission rates. The rate of viral transmission per minute of contact from an infected person in stratum *j* to a susceptible person in stratum *i* (where *i*, *j = 1,2,3,4,5*) was denoted by *λ_ϋ_*. The probability that transmission occurred during a contact of *d* minutes was 1 – exp {–*λ_ϋ_d}*. On each day of the simulated outbreak, the model calculated for each susceptible person the probability of becoming infected that day, based on the contacts made with all persons in each contact group. Consider a susceptible person *A* from stratum *i_A_* and a person *B* in one of *A*'s contact groups, Ψ *_Ajkl_* . Define *^y^_B_* = 0 if *B* was not infectious and *^y^_B_* = 1 if *B* was infectious. The probability that *A* escaped infection from *B* that day was exp {–*λ_ϋ_d_iAjkl_^γ^_B_*}. To remain uninfected, *A* must have escaped infection from all the members of all her/his contacts groups. Hence the probability that *A* became infected on this day was: *^P^*^(inf) =^ ^1 –^ ^П^*^k^*^П^*^j^*^П^*^BЄψ^_Ajkl_* exp {–*λ_ϋ_d_iAjkl_^γ^_B_*} . This probability was compared with a random number, *r*, drawn from the interval [0,1]. The person *A* became infected if *r* < *P*(inf).

Each newly infected person entered a latent period, at the conclusion of which the person became infectious to others, based on values estimated by Elveback et al. (*21*). We assumed that the probability of symptoms developing, given influenza infection, was 0.67 and that an infected person who did not become ill was 50% less infectious than one who did. An ill person with severe symptoms withdrew to home, made contacts only with household members, and the duration of these contacts was decreased by 50%. We assumed that in 50% of adults and 75% of children severe symptoms developed and the person withdrew. An ill person could require hospitalization or die from influenza complications. The probabilities of hospitalization and death were determined on the basis of the distribution of age-specific hospitalizations and deaths in an average seasonal (nonpandemic) influenza season (*22**–**24*) and on the total hospitalization and death rates expected in a pandemic that is similar to the Asian influenza pandemic of 1957–58, for which the overall illness attack rate was estimated at 0.33, with an influenza death rate of 0.58/1,000 persons (*25*). A list of the initial settings of all the parameters used in these models is provided below.

The simulated epidemic started with a small number of infective persons. The transmission process continued until no further infected persons remained in the community. At the end of each simulated epidemic, the program determined the proportion of persons who became ill as well as the proportions of hospitalizations and deaths in the community. We ran 200 simulations and calculated the means of the above 3 proportions over these simulated epidemics.

### Baseline Values of Parameters

#### **A. Influenza-related Parameters (based on Longini et al. [** 25 **])**

Pandemic illness rates by age group (used for calibration of transmission rates and contact parameters): 0–4 years, 36%; 5–18 years, 62%; 19–64 years, 25%; >65 years, 21%; overall rate, 33%.

Probability of illness given infection = 0.67.

Relative infectiousness of infected persons who do not become ill = 0.50.

Rate of withdrawal due to "severe" symptoms: in children, 0.75; in adults, 0.50.

Relative contact rate when withdrawn due to "severe" symptoms = 0.50.

#### B. Transmission Rates

We assumed that the transmission rate (transmission probability per 1 minute of contact) might vary by the ages of the infected and susceptible persons but not by the mixing group or by weekday versus weekend day. The values of the transmission rates, which are presented in Table A1, were determined in a calibration process so that the above illness attack rates were obtained.

#### C. Probabilities of Hospitalization and Death, given Illness, by Age Group

For the purpose of estimating the hospitalization and death probabilities, we used 9 age groups. We started with data on influenza-related hospitalization and death rates for an average seasonal influenza epidemic (22–44). We then adjusted these rates so that we obtained the predicted overall rates for a pandemic (247 and 70 per 100,000, respectively, based on Meltzer et al. [26]). To determine the conditional probabilities for ill persons we divided these rates by the expected pandemic illness rates listed in section A. The conditional probabilities are presented in Table A2.

#### D. Contact Frequencies and Durations

##### D.1. Persons Who Reside at Home

Four age strata are included in the simulation models: 0–4; 5–18; 19–64; and >65 years. There are 5 types of mixing groups: households, daycare centers, schools, workplaces, and the community (for contacts of long-term care facility [LTCF] residents, see section D.2). For a given mixing group and type of day, and for each combination of 2 strata (*i*, *j*) we needed to determine: (i) the number of persons from stratum *j*contacted in 1 day by a person from stratum *i*, *c*_ϋ_ , and (ii) the average total duration per day (in minutes) of all the contacts with 1 person, *^d^ϋ*. These numbers are symmetric: *c_ji_* - *c*_ϋ_ and *d_ji_* = *^d^ϋ* .

##### D.1.a. Weekdays

Contacts in the household: We assumed that each member of the household contacted every other member, so we did not specify *c*_ϋ_'s. Table A3 presents values for the *^d^ϋ*'s.

Contacts in daycare centers: *^c^*_11_ = 6, *^d^*_11_ = 60. All other contact parameters are zero.

Contacts in schools: *^c^*_22_ = 10, *^d^*_22_ = 120 . All other contact parameters are zero.

Contacts in workplaces: *^c^*_33_ = 10, *^d^*_33_ = 120. All other contact parameters are zero.

Contacts in the community: Table A4 presents the values of (*c*_ϋ_ ,*^d^ϋ*). For simplicity, we assume that no contacts occur between children and adults in the community.

##### D.1.b. Weekend days

On a weekend day, contacts are made only in households and in the community. The weekend values of the *^d^ϋ*'s in households and in the community are twice the corresponding weekday values. The community weekend values of the *c*_ϋ_'s are twice the corresponding weekday values.

#### D.2. LTCF residents

Each LTCF resident made contacts with 4 other residents for an average of 120 minutes (on weekdays and on weekend days) and with 2 members of the LTCF staff for 120 minutes (weekdays and weekend days). In addition, this person has contact with 1 family member for 60 minutes on weekdays and with 2 family members for 120 minutes each on weekend days.

To illustrate the computation of the daily infection probabilities, we assume that person A is a susceptible school-aged child (stratum 2) who lives in a household with 2 parents (ages 19–64, stratum 3) and a younger preschool child (stratum 1). We now calculate the probability that A will become infected on a given weekday. For this illustration we make the simplifying assumption that every person with whom A makes contact is infectious on this day. Person A makes contact in the household, in his or her school and in the community.

In the household, A makes contacts lasting a total of *d*_21_ = 60 minutes (Table A3) with the preschool child. The per-minute transmission rate from infectious younger child to person A is *λ*_21 =_ 0.00062 (Table A1). Therefore the probability that A escapes infection from that child is exp(–0.00062 x 60) = exp(–0.0372) = 0.9635. Person A also makes contact with his or her parents. The total duration of the contacts with each parent is *d*_23_ = 120 minutes (Table A3), and the transmission rate from the infected parent is *λ*_23_ = 0.00053 (Table A1). Therefore, the escape probability from each parent is exp (-0.00053 x 120) = 0.9384. The probability that *A* escapes infection from all the household members is 0.9635 x 0.9384^2^ = 0.8485.

In the school, person A makes contact with 10 other schoolchildren (*^c^*_22_ (school) = 10 , where the total duration of the contacts that each child makes is 120 minutes (*^d^*_22_ (school) = 120). The per-minute transmission rate is *^λ^*_22_ = 0.00061. Therefore the escape probability from all school contacts is [exp(-0.00061 x 120)]^10^ = 0.4809.

In the community, person A makes contact with 1 preschool child (*^c^*_21_(community) = 1 , Table A4) lasting a total of 30 minutes ( *^d^*_21_(community) = 30, Table A4), and with 2 school-aged children ( *^c^*_22_(community) = 2), for a total of 60 minutes each (*^d^*_22_(community) = 60. The per-minute transmission rates from the preschool child and from each school-aged child are *^λ^*_21_ =0.00062 and *^λ^*_22_ =0.00061, respectively. Hence the escape probability from all the community contacts is [exp(-0.00062 x 30)] x [exp(-0.00061 ^x 60^)]^2^ = 0.9123.

Thus, the overall probability that person A becomes infected on this day 1- 0.8485 x 0.4809 x 0.9123 = 0.6277 is. (This very high daily probability of infection is the result of the assumption that all the persons with whom A makes contact on this day are infectious.) To determine if *A* actually becomes infected, a random number between 0 and 1 is generated, and if this number does not exceed 0.6277, then the simulation program determines that *A* becomes infected on this day.

## Appendix

**Table A1:** Transmission rates (λ_ϋ)_ from an infectious person in age group *j* to a susceptible person in age group *i*.

Age group of infectious (*j*), y	Age group of susceptible (*i*), y			
	0–4	5–18	19–64	>65
0–4	0.00059	0.00062	0.00033	0.00080
5–18	0.00058	0.00061	0.00033	0.00080
19–64	0.00057	0.00053	0.00032	0.00080
>65	0.00057	0.00054	0.00029	0.00102

**Table A2:** Age-specific conditional probabilities of hospitalization and death, given influenza infection

Age group, y	Hospitalization	Death
0–4	0.00810	0.00005
5–18	0.00091	0.00003
19–49	0.00227	0.00007
50–64	0.00907	0.00148
65–69	0.02442	0.00530
70–74	0.04125	0.00928
75–79	0.05539	0.01805
80–84	0.08816	0.03529
>85	0.15357	0.09583

**Table A3:** Duration of contacts with household members

Stratum	1	2	3	4
1	120	60	120	60
2	60	120	120	60
3	120	120	120	120
4	60	60	120	120

**Table A4:** Number of contacted persons and total duration of all contacts with 1 person in the community

Stratum	1	2	3	4
1	2, 60	1, 30	0	0
2	1, 30	2, 60	0	0
3	0	0	2, 60	2, 60
4	0	0	2, 60	2, 60
